# Abdominal Closure With Reinforcing Suture Decreases Incisional Hernia Incidence After CRS/HIPEC

**DOI:** 10.3389/jaws.2023.11188

**Published:** 2023-03-09

**Authors:** Charlotta Wenzelberg, Ulf Petersson, Ingvar Syk, Olle Ekberg, Peder Rogmark

**Affiliations:** ^1^ Department of Surgery, Skane University Hospital Malmö Sweden and Department of Clinical Sciences Malmö, Lund University, Lund, Sweden; ^2^ Department of Radiology Diagnostics, Skane University Hospital Malmö Sweden and Department of Translational Medicine Malmö, Lund University, Lund, Sweden

**Keywords:** incisional hernia, abdominal closure, reinforcing suture, reinforced tension line suture, hyperthermic intraperitoneal chemotherapy

## Abstract

**Background:** Cytoreductive surgery (CRS) and hyperthermic intraperitoneal chemotherapy (HIPEC) entails several risk factors for incisional hernia (IH). A few reports available showing incidences between 7% and 17%. At our institution fascia closure has been performed in a 4:1 suture to wound length manner, with a continuous 2-0 polydiaxanone suture (PDS-group) or with a 2-0 polypropylene suture preceded by a reinforced tension line (RTL) suture (RTL-group). Our hypothesis was that these patients might benefit from reinforcing the suture line with a lower IH incidence in this group. The aim was to evaluate the 1-year IH-incidence of the two different closures.

**Methods:** Patients eligible for inclusion were treated with CRS/HIPEC between 2004 and 2019. IH was diagnosed by scrutinizing CT-scans 1 year ±3 months after surgery. Additional data was retrieved from clinical records and a prospective CRS/HIPEC-database.

**Results:** Of 193 patients, 129 were included, 82 in the PDS- and 47 in the RTL-group. RTL-patients were 5 years younger, had less blood loss and more frequent postoperative neutropenia. No difference regarding sex, BMI, recent midline incisions, excision of midline scars, peritoneal cancer index score, complications (≥Clavien-Dindo 3b), or chemotherapy. Ten IH (7.8%) were found, 9 (11%) in the PDS- and 1 (2.1%) in the RTL-group (*p* = 0.071).

**Conclusion:** An IH incidence of 7.8% in patients undergoing CRS/HIPEC is not higher than after laparotomies in general. The IH incidence in the PDS-group was 11% compared to 2% in the RTL-group. Even though significance was not reached, the difference is clinically relevant, suggesting an advantage with RTL suture.

## Introduction

Cytoreductive surgery and hyperthermic intraperitoneal chemotherapy (CRS/HIPEC) is an extensive procedure for treating different peritoneal surface tumor spread as mesothelioma, pseudomyxoma peritonei (PMP) and gastrointestinal malignancies (peritoneal carcinomatosis, PC) (1). These patients face numerous risks for early postoperative complications related to advanced malignant disease, extensive surgery and intra-operative chemotherapy. With improved outcome and long-term survival, long-term sequelae become increasingly important to prevent.

IH is one of the most common complications after abdominal surgery with varying incidences. In a large meta-analysis by Bosanquet (2), incidences between zero and 36% were found and in the randomized controlled STITCH trial, comparing large-bite to small-bite closure, the IH-incidences at 1 year were 21% and 13%, respectively (3). IH causes morbidity, reduced quality of life, and need for reconstructive surgery (2-6).

Patients treated with CRS/HIPEC have several factors associated with increased risk for developing IH. They have often undergone earlier midline laparotomies; CRS/HIPEC surgery requires long midline incisions, often combined with excision of any previous scars; they are exposed to long operation times and; they receive intraperitoneal chemotherapy, resulting in prominent intestinal swelling and increased intraabdominal pressure at closure as well as secondary immunosuppression and low postoperative albumin levels (2, 5, 7, 8, 9).

Until now, only a few articles on IH after CRS/HIPEC surgery are available, reporting IH incidences between 7% and 17%. Results so far are difficult to compare. The number of patients evaluated vary between 155 and 282 with follow-up times between 8 and 38 months. Furthermore, the criteria for inclusion, and the modality for IH diagnosis vary. The preconceived suspicion of high IH rates after CRS/HIPEC have so far not been verified. Within this group of patients, higher age, higher BMI, female gender, neoadjuvant chemotherapy and fascial dehiscence (FD) seem to be independent risk factors for developing IH (5, 10, 11).

The gold standard technique for fascial closure after midline incisions today is the small-bite 4:1 suture to wound length ratio technique described and evaluated by Milbourn (9), and recommended in the European Hernia Society guidelines on closure of abdominal wall incisions (12). In 2007, Hollinsky described the Reinforced Tension Line (RTL) technique for treating IH, and reported promising results (8). Agarwal evaluated the RTL-technique in patients with acute peritonitis and found significantly lower rates of fascial dehiscence (FD) with RTL-closure compared to standard closure (13). Recently Lozada-Hernández published results from a randomized controlled trial with 3-year follow-up comparing the RTL-technique to mass closure technique in 104 patients with high risk for IH development, and found significantly lower IH incidence after using RTL-closure (9.8% vs. 28.3%) (14). Even if the scientific basis for the RTL-technique still is frail, the theoretical basis is appealing and the technique has been used at our institution in situations where reinforcement of the incision line is desirable, and mesh reinforcement is deemed unsuitable.

Fascia closure after CRS/HIPEC-procedures has been performed in either of two ways at our institution. In the earlier period, a continuous 2-0 polydiaxanone (PDS) suture in a 4:1 manner was standard. Since 2016, a 2-0 polypropylene (PP) RTL-suture followed by a 4:1 closure with the same suture material, has been the predominant method.

We hypothesized that CRS/HIPEC-operated patients might benefit from reinforcing the suture line rendering a lower incidence of IH compared to patients closed with the standard continuous PDS suture. The primary aim of this study was to evaluate and compare the 1-year computed tomography (CT) detected IH-incidence of the two different closure techniques. Secondary aims were to evaluate possible risk factors for IH and to describe the incidence of fascial dehiscence (FD).

## Materials and Methods

### Study Design and Aim

This is a retrospective, single-centre study from the Department of Surgery, Skåne University Hospital, Malmö, Sweden. Patients treated with CRS/HIPEC between September 2004 and September 2019 through a midline laparotomy, were eligible for inclusion. The primary aim was to evaluate the IH incidence with CT performed 12 ± 3 months after surgery and to compare the IH incidences between the two closure techniques. IH was defined according to the EHS definition (15) as “any abdominal wall gap with or without a bulge in the area of a postoperative scar, perceptible or palpable by clinical examination or imaging.” CT-scans were scrutinized for IH by three independent examiners (two surgeons and one radiologist). In case of discrepancy between the examiners’ interpretations, a discussion was carried out to reach consensus*.*

Patients closed in a different way than with the two techniques of interest or with existing midline mesh or hernia; patients deceased or re-operated within 9 months after surgery for any reason, and; patients not investigated with a CT scan 12 ± 3 months after surgery were excluded from the statistical analysis.

Secondary aims were to evaluate possible risk factors for IH and to describe the incidence of FD.

The RTL-technique was used in a few patients 2013–2016 and from 2017, it has been the predominant method. The study thereby reflects two fascia closure techniques but, to some extent, also two time periods of CRS/HIPEC-surgery at our institution.

### Fascia Closure Techniques

The fascia closure was performed in a 4:1 manner with either a continuous 2-0 PDS suture according to the description by Millbourn et al. (9) (PDS-group) or with a 2-0 PP suture, preceded by a RTL-suture of PP according to Hollinsky et al (8) (RTL-group).

The 4:1 technique has been the standard fascia closure technique at our department for many years. Data on the SL/WL ratio is however, not routinely recorded. In this series, the same surgeon has either performed or supervised all operations and fascia closure has been performed in the same way, with or without the RTL suture. PDS is our standard suture for fascial closure if no prophylactic measure for prevention of IH is deemed necessary. The RTL technique was initially introduced at our department as an alternative to the use of prophylactic mesh, e.g., in emergency surgery in high risk patients, and a non-absorbable material (PP) was chosen to mimic the mesh material.

**FIGURE 1:**
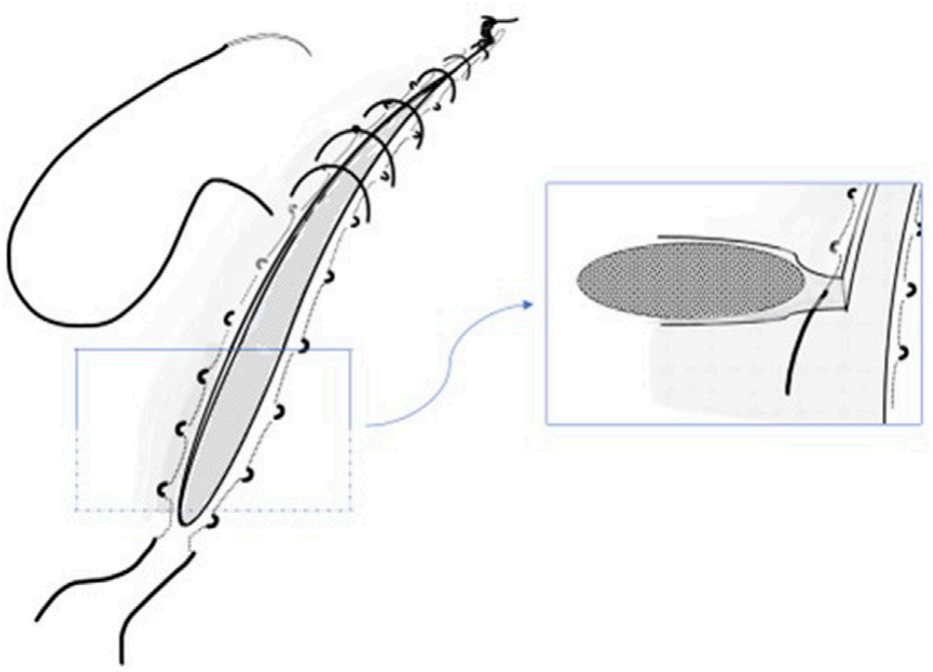
Schematic sketch depicting the Reinforced Tension Line (RTL) technique in abdominal fascial closure.

The RTL-suture is, according to the original description, placed within the condensed linea alba when possible. This means that the RTL suture is threaded within the fascia, parallel to the incision on both sides, starting and finishing at the caudal end of the incision, where the suture-ends are left untied at first. Since many patients have undergone earlier laparotomies or previous midline incision for the same malignancy, excision of scar tissue and linea alba many times leads to incision of both the anterior and posterior rectus fascia. In case the rectus sheath is opened, and the muscle exposed, the RTL suture is used to close the fascial layers. The following continuous 4:1 closing suture is placed just outside and including the RTL-suture in every stitch. Mass closure including muscle was not intended. Finally, the RTL suture is tied (Figure 1).

### Data Variables and Ethical Approval

Patient data were retrospectively retrieved from clinical records and from a prospective CRS/HIPEC-database. Retrieved data variables are shown in Tables 2, 3. The carcinomatosis was staged by use of the Peritoneal cancer index score (PCI), described by Jaquet and Sugarbaker (16). The completeness of surgical extirpation of cancer deposits was classified by use of the Completeness of cytoreduction score (CC-score) introduced by Sugarbaker (17), where CC0 is defined as no remnant disease, CC1 as remaining nodules less than 0.25 cm, CC2 nodules as 0.25–2.5 cm and CC3 as nodules exceeding 2.5 cm or confluent. Postoperative complications were classified according to the Clavien-Dindo classification from 2004 (18).

The Swedish Ethical Review Authority (Dnr 2020-03504) approved the study. In this retrospective study based on CT-scans and data from clinical records, informed consent was not required.

### Statistical Analyses

Data was analysed using IBM SPSS Statistics version 26.0.0.1. Continuous variables were expressed as mean with standard deviation (SD) or as median with interquartile range (IQR). Comparison between groups was calculated with Student’s t test, Pearson’s chi-square test or Fisher’s exact test, as appropriate. A p-value of ≤0.05 was considered statistically significant.

## Results

**FIGURE 2:**
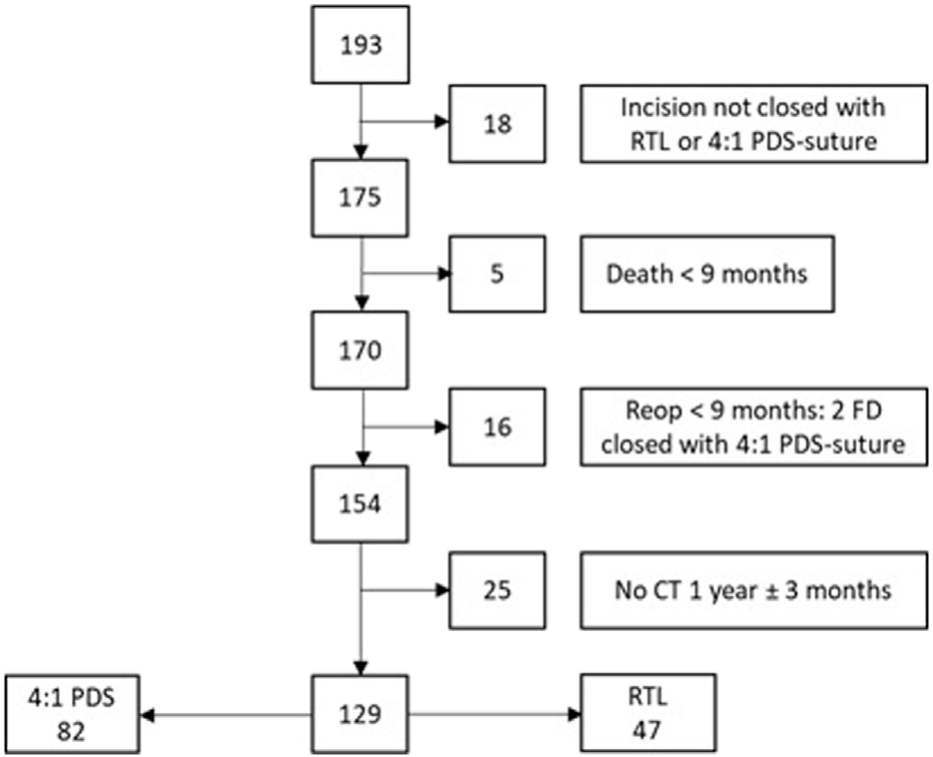
Study flow chart.

A total of 193 CRS/HIPEC-treated patients were identified, of which 64 patients were excluded, leaving 129 patients for analysis: 82 in the PDS-group and 47 in the RTL-group (Figure 2).

### Patient Baseline Characteristics

The indication for CRS/HIPEC-treatment was colorectal and appendix cancer in 87% of the cases (Table 1). We found no significant differences for patient baseline characteristics besides RTL-patients being 5 years younger (Table 2).

**TABLE 1:** Indications for CRS/HIPEC.

CRS/HIPEC indication, n (%)	
Colon cancer	64 (49.6)
Appendix cancer	33 (25.6)
Rectal cancer	15 (11.6)
Peritoneal pseudomyxoma	12 (9.3)
Small bowel cancer	3 (2.3)
Fallopian tube cancer	1 (0.8)
Malignant mesothelioma	1 (0.8)

**TABLE 2:** Preoperative characteristics.

	Total *n* = 129	RTL *n* = 47	PDS *n* = 82	*p*-value
Preoperative characteristics				
Female, n (%)	67 (52%)	23 (49%)	44 (54%)	0.605
Age, mean (SD)	57 (26.8)	54 (13.2)	59 (12.3)	0.026
ASA 1	20 (15.5%)	7 (14.9%)	13 (15.9%)	
ASA 2	77 (59.7%)	31 (66.0%)	46 (56.1%)	
ASA 3	32 (24.8%)	9 (19.1%)	23 (28.0%)	0.260*
Body Mass Index, BMI	26.0 (4.1)	26.3 (4.5)	25.8 (3.9)	0.573
Obesity BMI ≥30 kg/m^2^	23 (18.1%)	11 (23.4%)	12 (15.0%)	0,235
Chronic obstructive pulmonary disease, COPD, n(%)	8 (6.2%)	3 (6.4%)	5 (6.1%)	0.948
Ischemic coronary heart disease, ICHD	35 (27.1%)	10 (21.3%)	25 (30.5%)	0.257
Diabetes Mellitus, DM	11 (8.5%)	3 (6.4%)	8 (9.8%)	0.509
Immunosuppression therapy	6 (4.7%)	1 (2.1%)	5 (6.1%)	0.303
Hemoglobin (g/L)	132 (17.8)	133 (19.5)	131 (16.8)	0.599
Serum creatinin	72 (14.3)	71 (14.7)	73 (14.1)	0.447
Serum albumin (g/L)	38 (5.6)	39 (5.5)	38 (5.7)	0.203
Earlier midline laparotomy	38 (40.9%)	13 (37.1%)	25 (43.1%)	0.533
Midline laparotomy within 8 weeks^†^	36 (27.9%)	12 (25.5%)	24 (29.3%)	0.901
Neoadjuvant chemotherapy	32 (24.8%)	12 (25.5%)	20 (24.4%)	0.855

ASA 1 + ASA 2 vs ASA 3.

^†^ Laparotomy associated with the present malignancy, within 8 weeks before CRS/HIPEC-surgery.

### Peri-/Postoperative Characteristics and Incisional Hernia Incidence

Perioperative and postoperative findings, measures and complications were similar between groups. The only difference found was less blood loss and more frequent neutropenia in the RTL-group. Ten patients (7.8%) were diagnosed with an IH: 9 (11%) in the PDS- and 1 (2.1%) in the RTL-group (*p* = 0.071) (Table 3). Two cases of FD were noted, both in the PDS group.

**TABLE 3:** Perioperative and postoperative characteristics and incisional hernia incidence.

	Total *n* = 129	RTL *n* = 47	PDS *n* = 82	*p*-value
Perioperative findings				
Peritoneal cancer index, PCI, mean (SD)	11 (8.1)	9 (7.9)	12 (8.1)	0.087
Resection of midline scar, n (%)	93 (73.2)	32 (68.1)	61 (76.3)	0.316
Duration of surgery (min), mean (SD)	606 (181.4)	591 (170.3)	616 (188.8)	0.472
Blood loss (mL), mean (SD)	1156 (1165.6)	800 (695.6)	1394 (1347.1)	0.007
Complete Cytoreduction				0.412^*^
CC0	117 (95.9%)	45 (97.8%)	72 (94.7%)	
CC1 + CC2	5 (4.1%)	1 (2.2%)	4 (5.3%)	
Postoperative outcomes				
Neutropenia (WBC<1x109/L), n (%)	14 (10.9%)	9 (19.1%)	5 (6.1%)	0.022
Complication severity				0.717^**^
Clavien-Dindo 1	44 (34.1%)	14 (29.8%)	30 (36.6%)	
Clavien-Dindo 2	61 (47.3%)	24 (51.1%)	37 (45.1%)	
Clavien-Dindo 3a	17 (13.2%)	6 (12.8%)	11 (13.4%)	
Clavien-Dindo 3b	4 (3.1%)	1 (2.1%)	3 (3.7%)	
Clavien-Dindo 4a	3 (2.3%)	2 (4.3%)	1 (1.2%)	
Clavien-Dindo 5	0 (0.0%)	0 (0.0%)	0 (0.0%)	
Adjuvant chemotherapy	69 (53.5%)	24 (51.1%)	45 (54.9%)	0.901
Incisional hernia development				
CT-verified hernia	10 (7.8%)	1 (2.1%)	9 (11%)	0.071

^*^CC0 vs CC1 + CC2.

^**^Claven-Dindo 1–3a vs Claven-Dindo 3b–5 (need for interventions in general anesthesia, ICU treatment or death).

### Risk Factor Assessment

Data was grouped according to IH status (IH and no IH) and the variables in Table 2, 3 were analysed. In univariate analysis the presence of cardiovascular disease was higher among patients developing an IH, *p* = 0.024. No other differences were found. No multivariate analysis was carried out due to the few IH in this study.

## Discussion

This retrospective study compares IH incidences for gold-standard 4:1 PDS closure to RTL-suture plus 4:1 closure with PP, in patients treated with CRS/HIPEC for carcinomatosis. The total CT-detected IH incidence at 1 year was 7.8%. Nine of the IH were found in the PDS group and only one in the RTL group. The results represent a clinically relevant, albeit not statistically significant, difference between the closure techniques. In addition to these findings of IH at 1 year, it is noteworthy that there were two patients suffering a FD, both in the PDS-group.

IH is the most common long-term complication after abdominal surgery. IH causes increased morbidity, reduced quality of life, and need for further surgical interventions (2, 5, 6), sometimes as emergency operations due to obstruction, incarceration, and strangulation (4). Patients treated with CRS/HIPEC exhibit several factors associated with increased risk for IH (2, 5, 8, 9) and are thereby believed to develop IH to a greater extent. However, from the few studies on IH following CRS/HIPEC, incidences of 7%–17% are reported (5, 7, 10, 11) which do not exceed incidences after laparotomies in general (2, 3).

In this study CT-diagnosed IH at 1-year was found in 7.8% overall. 11% in the PDS-group is in the range of previous reports whilst 2% in the RTL-group stands out as low. Comparison of incidences between available studies must be made with caution due to diverting study protocols, different follow-up times and modality for IH diagnosis. CT has a higher sensitivity for IH diagnosis than physical examination (19) but will, on the other hand, certainly detect some clinically irrelevant IH. Even if uncertainty remains as to the real IH incidence following CRS/HIPEC procedures, it seems as if the IH risk is not elevated as could be expected, but rather surprisingly low.

In earlier studies on IH after CRS/HIPEC operations, higher age, higher BMI, female gender, FD, neoadjuvant chemotherapy and HIPEC in ovarian cancer have been shown to be independent risk factors for developing IH (5, 7, 10, 11). In the univariate analysis, none of the above mentioned variables were found to be risk factors for IH. The only risk factor for IH development found in univariate analysis was the presence of cardiovascular disease. The few IH in this study did not allow for a multivariate analysis and whether cardiovascular disease is an independent risk factor was thus not possible to investigate (20).

There seem to be factors balancing the effect of factors associated with increased IH incidences present in CRS/HIPEC patients. We do not have one plausible explanation, but the patients assessed for a CRS/HIPEC procedure are thoroughly evaluated and, beside their malignancy, must be relatively healthy to be considered for such extensive and complication prone surgery. In the available studies the median age varied between 52 and 60 years, median BMI between 24 and 29, presence of cardiopulmonary disease and diabetes mellitus was reported in two studies as 26% and 33%, and 8% and 10%, respectively. In four of the five studies ASA were reported and more than 70% of the patients were classified as ASA 1 or 2. These findings indicate that the CRS/HIPEC patients reported so far may have less preoperative IH risk factors than the general laparotomy patient. CRS/HIPEC are highly specialized procedures performed or supervised by a few very experienced surgeons. All articles are single tertiary referral centre reports, which ensures conformity of the surgical strategy within the studies, including the abdominal closure technique. Another theoretical explanation to the relatively low IH incidence is the extensive formation of adhesions following peritonectomy and chemotherapy, which may distribute increased intraabdominal pressure more evenly to the abdominal wall and thereby prevent focused tension on the closed midline incision.

Despite the fact that IH incidence after CRS/HIPEC so far has not been shown to be increased compared to laparotomies in general, it is of importance to prevent the morbidity linked to IH in this group of heavily burdened patients. Mesh is successfully used for reinforcement of the suture line after laparotomy in patients with high risk for IH (21). Use of mesh in CRS/HIPEC patients might imply an increased risk for wound complications and delayed start of adjuvant chemotherapy (22). Reinforcing the suture line with a RTL suture as in this study, is far less evaluated (8, 13, 14) but is far less extensive and does not imply the same risks as mesh reinforcement and is thereby an appealing alternative worth evaluating.

FD seems to be more frequent after CRS/HIPEC than after laparotomies for other causes, with reported incidences of 4%, 5.3% and 7.1% (5, 10, 23). The consequences of a FD are more serious than for an IH and thereby of even greater importance to prevent. In this study we only found 1.6% FD where both patients belonged to the PDS-group. It takes a much larger cohort to find out if the use of an RTL-suture offers protection against fascial dehiscence.

There are some weaknesses with this study. The drawbacks of a retrospective design are to some extent counteracted by data retrieval from a prospective database and by the standardized surgical technique achieved by the participation of one senior surgeon at all operations. We have not had the intention to describe the true IH incidence over time but rather at 1 year, a time when approximately half of the surviving patients are likely to have developed an IH. The cumulative incidence is thereby for sure underestimated. CT-scans were made for cancer treatment follow-up without Valsalva manoeuvre, which also may underestimate IH incidence to some extent. The RTL-technique was mainly used from 2017 and onwards which reflects the latter period of CRS/HIPEC operations at our department, and may thereby reflect increased procedural skill, possibly affecting the results. The use of different suture materials, i.e., PP in the RTL-group and PDS in the PDS-group has a historical explanation at our department. The RTL technique was initially introduced as an alternative to the use of prophylactic mesh, e.g., in emergency surgery in high risk patients, and a non-absorbable material (PP) was chosen to mimic the mesh material. Besides the RTL-suture, the choice of PP may have contributed to the better outcome for this group.

We find the study results encouraging and the RTL plus 4:1 closure with non-absorbable suture has become standard for fascial closure in patients operated for peritoneal carcinomatosis with CRS/HIPEC at our institution.

## Data Availability

The raw data supporting the conclusion of this article will be made available by the authors, without undue reservation.
